# Air, Water, and Exercise

**Published:** 1857-08

**Authors:** 


### AIR, WATER, AND EXERCISE,

The three greatest medicines known, and the best;—for when employed in moderation and in proper proportions, they give health to the body, vigor to the mind, and lovingness to the heart. They brighten the eye, quicken the intellect, and elevate the soul. In their train follow a sharp appetite, good digestion, and sound sleep. The blood is purified, the strength is renewed, and the whole physical man is invigorated. They plant perennial roses on the cheek of beauty, add largely to the powers of endurance in mature life, and give to old age the bodily agility of younger years, with the kindliness of childhood.

